# Vaccarin Ameliorates Doxorubicin-Induced Cardiotoxicity via Inhibition of p38 MAPK Mediated Mitochondrial Dysfunction

**DOI:** 10.1007/s12265-024-10525-7

**Published:** 2024-06-17

**Authors:** Xin Shi, Yang Cao, Hongyu Wang, Qi Zhao, Cong Yan, Shengzhu Li, Ling Jing

**Affiliations:** Department of Cardiology, First Affiliated Hospital of Harbin Medical University, 23 Youzheng Street, Nangang Qu, Harbin, 150001 Heilongjiang China

**Keywords:** Vaccarin, Doxorubicin, Cardiotoxicity, Antioxidant, p38 MAPK, Mitochondrial dysfunction

## Abstract

Doxorubicin is a frequently used chemotherapeutic agent for treating various malignancies. However, it leads to severe cardiotoxic side effects, such as heart failure, and elevates the risk of sudden cardiac death among cancer patients. While oxidative stress has been identified as the primary cause of doxorubicin-induced cardiotoxicity, therapeutic antioxidant approaches have yielded unsatisfactory outcomes. The aim of this study is to explore the therapeutic potential of vaccarin, an active flavonoid glycoside extracted from traditional Chinese herbal agent Semen Vaccariae, in doxorubicin-induced cardiotoxicity. We observed that vaccarin significantly ameliorates doxorubicin-induced heart dysfunction in mouse model and suppresses oxidative stress mediated cell apoptosis via specifically inhibiting the activation of p38 MAPK pathway. In vitro, we observed that vaccarin alleviates doxorubicin-induced mitochondrial membrane depolarization and ROS generation in H9c2 cell, but the p38 MAPK agonist anisomycin reverses these effects. Our findings provide a promising natural antioxidant to protect against DOX-induced cardiotoxicity.

**Graphic Abstract:**

**Figure:**
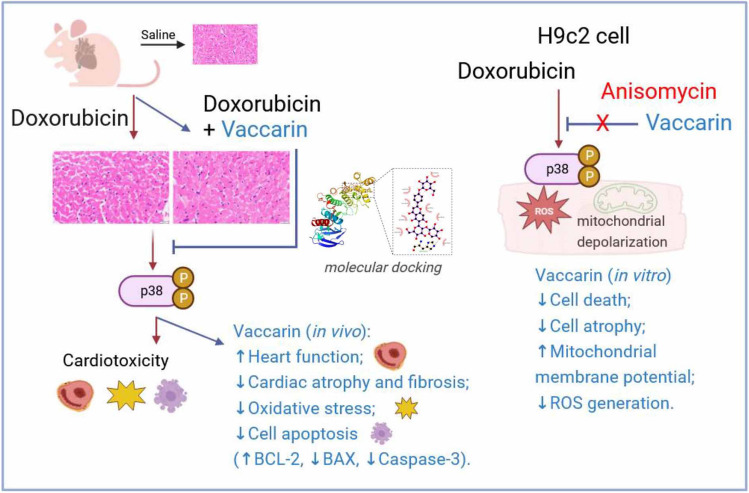
The new working model of Vaccarin Ameliorates Doxorubicin-Induced Cardiotoxicity via Inhibition of p38 MAPK Mediated Mitochondrial dysfunction

**Supplementary Information:**

The online version contains supplementary material available at 10.1007/s12265-024-10525-7.

## Introduction

Anthracycline drug doxorubicin (DOX) is an effective and commonly used chemotherapeutic agent for various malignancies [1]. However, it causes serious cardiotoxic adverse effects, including heart failure, and increases risks of cardiac sudden death among cancer patients, which impedes its wide clinical application. Accumulated research has been conducted, and the results identified that oxidative stress is the major cause of DOX cardiomyopathy. More specifically, a substantial generation of free radicals, impaired mitochondrial function, alterations in iron regulatory protein, increased intracellular calcium levels, nuclear translocation of p53, and subsequent destruction of subcellular structure, as well as apoptosis upon DOX accumulation ultimately can contribute to cardiomyopathy [2]. Nevertheless, therapeutic antioxidant approaches have yielded disappointing results. So far, dexrazoxane (DEX) is still the only FDA approved medicine for preventing anthracycline-induced cardiactoxicity by chelating iron to decrease the generation of ROS, thus preventing ROS-induced cardiomyocyte apoptosis [3]. However, serious challenges sustain since myelotoxicity is the frequently encountered side effect of dexrazoxane, which is very similar to the side effect of anthracyclines [4]. Therefore, discovering alternative natural antioxidants to protect against DOX-induced cardiotoxicity is in urgent need.

Vaccarin (VAC), an active flavonoid glycoside extracted from Semen Vaccariae, is frequently used in traditional Chinese medicine to promote blood circulation [5]. Recent studies recognized that VAC significantly stimulates endothelial cell proliferation in association with neovascularization [6, 7] and wound healing [8]. Multiple pathways, including activation of AKT and ERK signaling [6], activation FOXP2/AGGF1 [8] and FGF-2/FGFR1 [7], have been identified that mediate these effects. In addition, VAC has numerous protective effects on type 2 diabetes. These include ameliorating insulin resistance by activating the AMPK signaling pathway [9], improving intestinal barrier changes by inhibiting the ERK/MLCK signaling pathway [10], and antagonizing high glucose-induced endothelial dysfunction through regulating the ROS/AMPK/miRNA-34a/eNOS signaling pathway [11].

Besides, the protective effects of VAC on cells against oxidative stress-induced apoptosis have been demonstrated, and related mechanisms have been elucidated. For example, VAC protects human microvascular endothelial cells from apoptosis via attenuation of HDAC1 [12] and attenuates the human EA.hy926 endothelial cell oxidative stress injury through inhibition of Notch signaling [13]. Another study reported that VAC prevents ox-LDL-induced endothelial-to-mesenchymal transition (EndMT), inflammation and apoptosis by suppressing ROS/p38 MAPK signaling [14]. However, the effects of VAC on cardiomyocytes, in particular, in DOX-induced cardiotoxicity remain unknown.

To fill the knowledge gaps, this study aimed to investigate the effects of VAC on DOX-induced cardiotoxicity and its associated signaling pathway. Male mice were received VAC in addition to DOX treatment for 4 weeks to observe the heart function, morphology change, oxidative stress, cell apoptosis to determine the effects of VAC on DOX-induced cardiotoxicity. The expression of JNK, ERK, and P38 MAPK pathway related proteins were detected at the same time. In addition, H9c2 cardiomyocytes were pretreated with VAC followed with DOX administration to mimic the in vivo conditions, and P38 MAPK agonist, anisomycin was included to determine whether the pathway activation alters the VAC’s effects on DOX-induced cardiotoxicity. For the first time, this study identifies the effects and mechanisms of VAC on DOX-induced cardiotoxicity, aiding in the identification of therapeutic targets to enhance cardiac function in cancer patients post chemotherapy.

## Methods

### Animals

Male wild type (WT) (C57BL/6) mice (body weight, 16–20 g; age, 6 weeks) were purchased from Liaoning Changsheng biotechnology co., Ltd, China. The mice were housed in the animal facility under specific pathogen-free conditions. The experimental procedures were ethically approved by the Harbin Medical University Committee on Animal Care [15] and were carried out in accordance with the Regulations for the Administration of Affairs Concerning Experimental Animals approved by the State Council of China. After one week of adaptive feeding, thirty-five male mice were randomly divided into three groups. Each group of mice had free access to standard rodent diet and water. The experimental groups were as follows: (1) Saline only (Sham, *n* = 5), (2) doxorubicin only (DOX, *n* = 15), (3) doxorubicin followed by vaccarin (DOX + VAC, *n* = 15). Mice received 200ul saline or DOX at 5 mg/kg body weight via intraperitoneal (IP) injection once a week for four weeks. The third group additionally received VAC at 30 mg/kg body weight via IP daily for four weeks. All mice were euthanized on day 28 after anesthetizing with 1.25% avertin IP.

### Doppler Echocardiography and Sample Collction

In vivo Doppler echocardiography right before the end of the study. At day 28, five mice from each group were anesthetized using 1.25% avertin IP and were stabilized in the supine position, and M mode echo recordings from the parasternal long‑and short axes were collected. All measurements were subsequently analyzed using Vevo 770 software (Fujifilm VisualSonics Inc.). After euthanizing the mice, peripheral blood was collected in EDTA-coated tubes and centrifuged at 1,000–2,000 × g at 4 °C for 10 min. The resulting supernatant, designated plasma was carefully collected, aliquot and stored at -20 °C. Heart was pictured and weighed immediately after being taken out from the thoracic cavity and was divided into two parts. One was fixed in 4% paraformaldehyde solution, and then dehydrated, and embedded in paraffin. The other part was cut into small pieces followed by snap freezing in liquid nitrogen and stored at -80 °C.

### Histology

*H&E staining. *Stain the tissue Sects. (4 µm) with haematoxylin and eosin following the established protocol of our lab [15]. *Wheat germ agglutinin (WGA) staining.* Deparaffinize tissue Sects. (4 µm) tissue through three changes of xylene and hydrate using distilled water. Subsequent to rinsing, stain the slides with AF488-conjugate WGA (5 µg/ml, Sigma, US) in room temperature for 30 min. When labeling is complete, remove the labeling solution, and wash the slides three times in 2% PBST, then stain the slides in anti-fade mounting medium.

### ELISA

Homogenize the heart sample in 900 µl PBS per 0.1-g tissue. Next, centrifuge samples at 3000 × rpm for 20 min at 4 °C and take the supernatant for assay. Examine the brain natriuretic peptide (BNP) concentration in plasma and heart by mouse BNP ELISA kit (Elabscience, China) following the recommended protocol. Measure the OD value at 450 nm. Examine the activity of SOD in plasma and heart homogenate by SOD assay kit (Nanjing Jiancheng Biological Engineering Institute, China) based on the auto-oxidation of hydroxylamine. Measure the OD value at 550 nm. Examine the MDA concentration using MDA assay kit (Nanjing Jiancheng Bioengineering Institute) based on thiobarbituric acid (TBA) reactivity. Briefly, after mixing trichloroacetic acid with the plasma and homogenate and centrifuging, obtain the supernatant, and add TBA. Measure the OD value at 532 nm with a spectrophotometer. Examine the LDH release by LDH *Assay Kit (Abcam, US). Briefly, transfer 10 µl culture medium into new plate, add LDH reaction mix, and incubate for 30 min at room temperature. Measure the OD value at 450 nm.*

### TUNEL Apoptosis Assay

Immunohistochemistry for apoptotic cells was performed using the terminal deoxynucleotidyl transferase-mediated deoxyuridine triphosphate nick‐end labeling (TUNEL) method using the In Situ Apoptosis Detection Kit (Red, Elab Fluor® 647, China). Briefly, paraffin embedded mouse heart was treated with DNase I to fragment the DNA. DNA strand breaks showed intense fluorescent staining in DNase I treated sample (red). The cells were counterstained with DAPI (blue). The photos were taken with a confocal microscope.

### RNA Extraction and Quantitative Real-Time PCR (qRT-PCR)

Frozen heart tissue (50-100 mg) were dissociated in 700ul TRIzol reagent (Invitrogen) and total RNA was isolated using an RNeasy Plus Mini Kit (Qiagen). The quality and the concentration of RNA were measured by Nanodrop 2000 (Thermo Scientific). Two μg of total RNA was reverse transcribed into cDNA using a high capacity cDNA reverse transcription kit (Invitrogen). The mRNA expression levels of the Bcl-1, Bax, and caspase3 genes were determined by qRT-PCR using 2X ChamQ Universal SYBR qPCR Master Mix (TransGen Biotech, China). The primers sequences are Actin-F *5’-CACTGTCGAGTCGCGTCC-3’*, Actin-R*5’-TCATCCATGGCGAACTGGTG-3’*, Bcl-2-F *5’-GTGGATGACTGAGTACCTGAAC-3’*, Bcl-2-R *5’-GAGACAGCCAGGAGAAATCAA-3’*, Caspase3-F *5’-CGCGCACAAGCTAGAATTTATG-3’*, Caspase3-R *5’ GGACACAATACACGGGATCT-3’*, Bax-F *5’-GCTGAGGCAACTTCAACTG-3’*, Bax-R *5’-ATCAGCTCGGGCACTTTAG-3’*. Relative expressions of the gene were calculated with 2^ΔΔCt^ methods using Beta-actin as a reference gene.

### Western Blot

Homogenize the frozen heart tissues and cell extracts and extract total proteins in ice-cold RIPA lysis buffer (Sigma) with a protease inhibitor mix (Sigma). Next, apply equivalent amounts of samples (30ug/well) onto an SDS–Polyacrylamide gel. After SDS-PAGE, transfer the proteins to PVDF membranes and block the membranes in TBST with 5% non-fat milk for an hour. Then incubate the membranes in primary antibodies solution at 4 °C overnight followed by three times wash with TBST and incubate them in peroxidase (HRP)-conjugated secondary antibodies (Abcam) at room temperature for an hour. The primary and secondary antibodies are Bax(1:1000, abcam, #ab182733), Bcl-2 (1:200, Santa, #sc-7382), Caspase3 (1:750,Abclonal, #A2156), p38 (1:1000, Affinity, #BF8015), p-p38 (1:1000, Affinity, #AF4001), ERK1/2 (1:1000, CAT, #9102), p-ERK1/2 (1:1000, CAT, #9101), JNK (1:1000, CAT, #9252), p-JNK (1:1000, CAT, #9251), Goat anti-Rabbit IgG (HRP) (1:10,000,CAT, #7074). Use GAPDH antibody (1:10,000, CAT, #2118) as a loading control. Apply HRP substrate (Millipore) solution on the PVDF membranes after washing. Use ChemiDoc XRS + system (Bio-Rad) for imaging and Fiji for image processing and analyzing.

### Active Ingredients Retrieval and Molecular Targets Prediction of Vaccarin

Upload the CAS registry number of *VAC* (53,452–16-7) into TCMSP database [16] and retrieve active ingredients. In detail, We used oral bioavailability (OB) > 30 and drug-likeness (DL) > 0.18 as screening criteria, following the standards for identifying 'drug-like' compounds in traditional Chinese herbs [16, 17], and got the four major ingredients. Next, we acquired Canonical SMILES (Simplified Molecular Input Line Entry System, a chemical structure line annotation for entering and representing molecules of each active ingredient and got their protein targets using SwissTargetPrediction (http://www.swisstargetprediction.ch/) [18]. Finally, we removed the duplicates and used Uniport to convert potential targets to gene symbol(https://www.uniprot.org/).

### Molecular Docking Analysis

Ligand and protein preparation were following the established protocol. Obtain the 3D format of vaccarin from the PubChem compound database. Use ChemBio3D Ultra 14.0 to calculate the 3D structure. The ligands were processed with minimize energy module of MM2 procedure in Chem3D, and subsequently saved as “mol2” files. Meanwhile, obtain the 3D structures of p38 proteins from Protein Data Bank. Use Pymol2.3 for ligand separation and use AutodockTools-1.5.6., which can batch molecules, hydrogenate charges and convert formats, to transform ligand and proteins into “.pdbqt” files. Next, use POCASA 1.1 to predict binding sites and AutoDock Vina1.1.2 to execute docking simulations. The binding site on the MAPK11 was described by creating a grid box with the dimensions of center_x = 25.4, center_y = -7.9, center_z = 35.4; MAPK12 with the dimensions of center_x = 41.0, center_y = 74.7, center_z = 2.8; MAPK13 with the dimensions of center_x = -10.8, center_y = 9.4, center_z = -30.0; MAPK14 with the dimensions of center_x = -6.6, center_y = -6.8, center_z = -3.6; and with a grid spacing of 0.375 Å, and size of X: 60; Y: 60; Z: 60. Following the completion of the docking study, choose the ligand docked pose with the least binding energy.

### Cell Culture

The study was conducted on an adherent H9c2 line of rat embryonic cardiomyocytes (ATCC, Manassas, VA, USA). Cultures were grown on Dulbecco’s Modified Eagle Medium (DMEM, Sigma), supplemented with 10% fetal bovine serum and 100 U/mL penicillin/streptomycin (Thermo Fisher). Culture the cells at 37 °C in 5% CO2-air. Next, passage the cultures after achieving 70–80% confluence. Rinse the cells with PBS solution, and then release the cells with trypsin and EDTA solution (Thermo Fisher). The suspension of released cells was centrifuged at 1000 × RPM for 3 min. Split the cell at a 1:2 ratio into a new culture bottle and start treatments after 70–80% confluence is achieved.

### Cell Viability Assay

Measure the cell viability by Hoechst/PI viability kit (Nexcelom). Briefly, culture and treat the cells in 96 well plate. Prepare 5X master mix with 5 ml PBS, 40 µl PI and 8 µl Hoechst 33,342. After removing medium and rinsing with PBS, add 40 µl 5X master mix into well containing 160 µl PBS. Incubate the plate for 15 min at RT in the dark. After staining, the plate is ready for imaging by confocal microscope without washing.

### Measurements of Cell Size in H9c2 Cells

H9c2 cells were visualized by CellMask™ Deep Red Actin Tracking Stain and DAPI (Thermo Fisher). Briefly, culture and treat cells in six well plate. After removing medium and rinsing with PBS, fix the cells were in 4% paraformaldehyde solution at RT for 10 min followed by triple PBS washing. Add 2 ml actin tracking stains (5 µM); incubate the plate for 30 min at RT in the dark. Then add DAPI at 1X concentration and observe cell size using a fluorescence microscope. Analyze at least 50 cells in each experiment using ImageJ software.

### Measurements of Mitochondrial Membrane Depolarization in H9c2 Cells

Determine the mitochondrial health in H9c2 cells by JC-1 staining kit (Thermo Fisher). Following the protocol, stain the H9c2 cells with 10 µg/mL JC-1 for 30 min at 37 °C in the dark. Observe the J-aggregate and monomer in fluorescence microscope at 590 nm (red) and 529 nm (green), respectively.

### Measurements of and ROS Generation in H9c2 Cells

Measure the ROS levels in H9c2 cells with 2’, 7’-dichlorodihydrofluorescein diacetate (DCFH-DA, 10 µM) staining for 30 min at 37 °C in the dark. After triple PBS washing, take representative fluorescent images for each well using the green fluorescent protein (GFP) channel on a fluorescence microscope.

### Statistics

All data were analyzed using GraphPad Prism (GraphPad Software, Inc.). The data represent the mean ± SEM. For comparisons between three groups, the unpaired Student t test was used for evaluation. For comparisons between more than three groups, one way ANNOVA the unpaired Student t test was used for evaluation. Data shown were representatives of 2–3 independent experiments.

## Results

### Vaccarin Ameliorates DOX-induced Cardiotoxicity

**Fig. 1:**
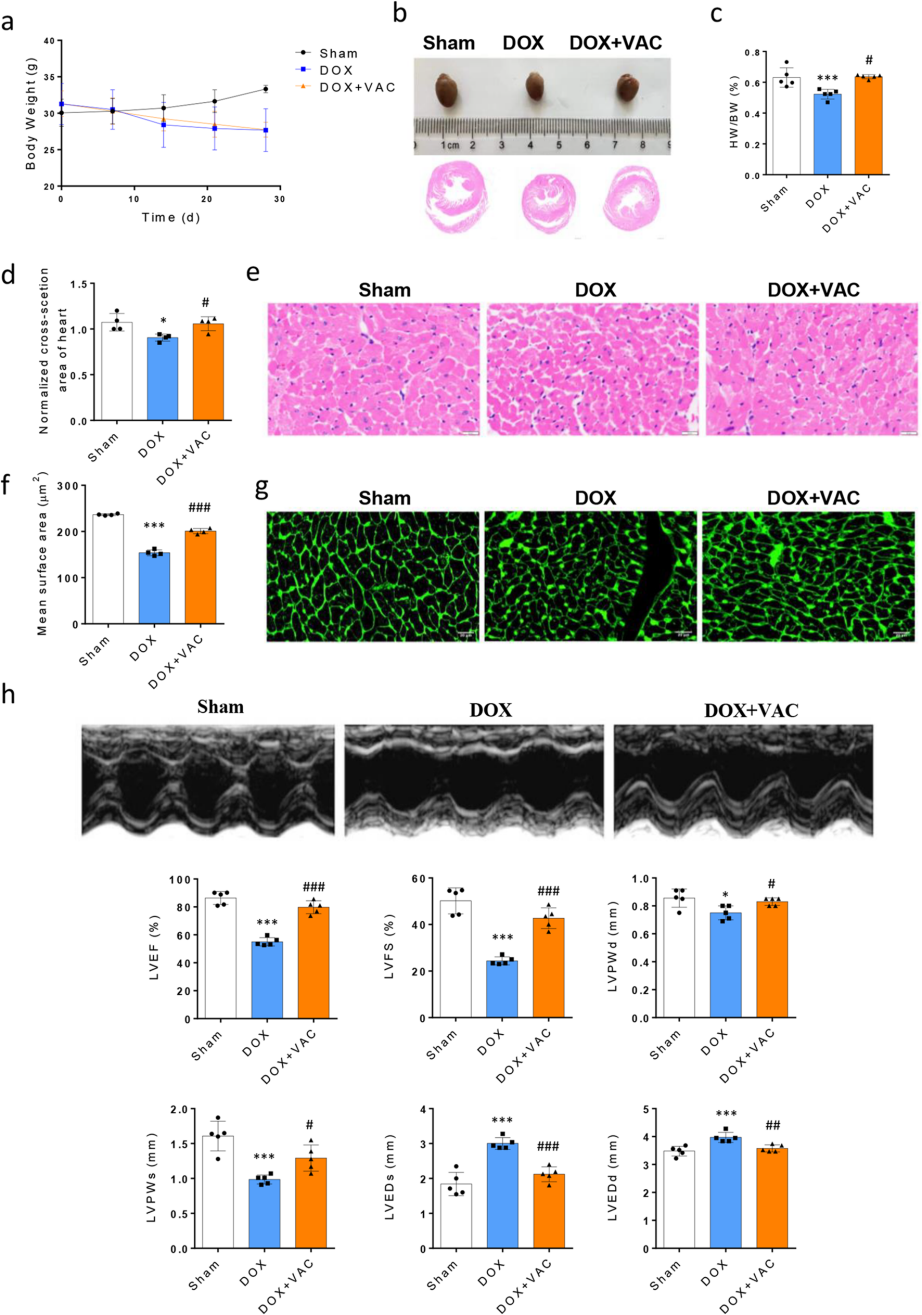
Vaccarin alleviates cardiac atrophy and improves left ventricular performance in a doxorubicin (DOX)-induced cardiotoxicity mouse model. **a** Body weight curve of the three groups of mice from the start of treatment to the end of the study. **b** Representative images of heart size and transversal cross section obtained by microscopic analysis (H&E stain) in sham, DOX treated, and DOX + Vaccarin (DOX + VAC) treated mice. **c**-**d** Bar graphs show the ratio between the heart-weight and body weight **c** and normalized cross-section area of heart in three groups **d**. **e** Representative images of cardiac morphology assessments by H&E stain (at 400 × magnification). **f**-**g** The average area of cardiomyocytes in three groups using of wheat germ agglutinin (WGA) stain (at 200 × magnification). **h** M-mode echocardiographic images were taken at the midventricular level (parasternal long axis) from three groups of mice right before the end of the study. Quantification of left ventricular ejection fraction (LVEF), left ventricular fractional shortening (LVFS), left ventricular posterior wall thickness in at diastole (LVPWd) and systole (LVPWs), Left ventricular end-systolic dimension (LVEDs) and end-diastolic dimension (LVEDd). Data are represented as the mean ± SEM (*n* = 5;* t* test, *p < 0.05, **p < 0.01, and ***p < 0.001 between groups of DOX and Sham. ^#^*p* < 0.05, ^##^*p* < 0.01, and ^###^*p* < 0.001 between groups of DOX + VAC and DOX)

Mice were treated with DOX (5 mg/kg, IP) weekly for 4 weeks to generate DOX-induced cardiotoxicity followed an established procedure [19, 20]. The total DOX dosage administered reached 20 mg/kg at the end of the treatment. To explore whether VAC can protect against DOX-induced cardiotoxicity, the third group of mice received VAC (30 mg/kg/d, IP) daily. This dose reproduces earlier findings that have significantly reduced hypertension, cardiovascular remodeling, and nephropathy in renovascular hypertensive rats [5, 21]. With the currently available large number of experimental models of DOX cardiomyopathy using small laboratory animals (mice and rats), no differences in pathological changes were observed in the modeling of DOX cardiomyopathy between female and male [22, 23]. Therefore, only male mice were used in this study. We measured the body weight weekly and found that mice challenged with DOX started to lose weight from day 7 (Fig. 1a) and exhibited smaller hearts at the termination time after 4 weeks. The group receiving DOX plus VAC treatment did not alleviate DOX-induced weight loose but significantly ameliorated DOX-induced heart shrinkage, which determined by size, heart weight/body weight ratio, and cross-section area (Fig. 1b-d). Cardiac morphology assessments by H&E staining also showed that VAC improved DOX-induced myocardial disarray and atrophy (Fig. 1e). The average area of cardiomyocytes assessed by wheat germ agglutinin (WGA) stain was larger in DOX + VAC treated mice (200.8 ± 2.8 µm^2^) than in DOX alone treated mice (153.8 ± 3.2 µm^2^) (Fig. 1f-g). In addition, echocardiography in mice revealed that DOX significantly decreased mouse left ventricular ejection fraction (LVEF, 55 ± 1.3%), left ventricular fractional shortening (LVFS, 24.4 ± 0.8%), left ventricular posterior wall thickness in at diastole (LVPWd, 0.75 ± 0.02 mm), and systole (LVPWs, 0.99 ± 0.03 mm), increases Left ventricular end-systolic dimension (LVEDs, 3.0 ± 0.07 mm), and end-diastolic dimension (LVEDd, 3.97 ± 0.08 mm). VAC treatment significantly restored the DOX-induced impairments of LVEF (79.8 ± 2.1%), LVFS (42.7 ± 2%), LVPWd (0.83 ± 0.01 mm), LVPWs (1.29 ± 0.08 mm), and decreases LVEDs (2.12 ± 0.1 mm), and LVEDd (3.58 ± 0.06 mm) (Fig. 1h). These data suggest that VAC alleviates cardiac atrophy and improves left ventricular performance in DOX-induced cardiotoxicity mouse model.

### Vaccarin Ameliorates DOX-induced Oxidative Stress and Cardiomyocyte Apoptosis

**Fig. 2:**
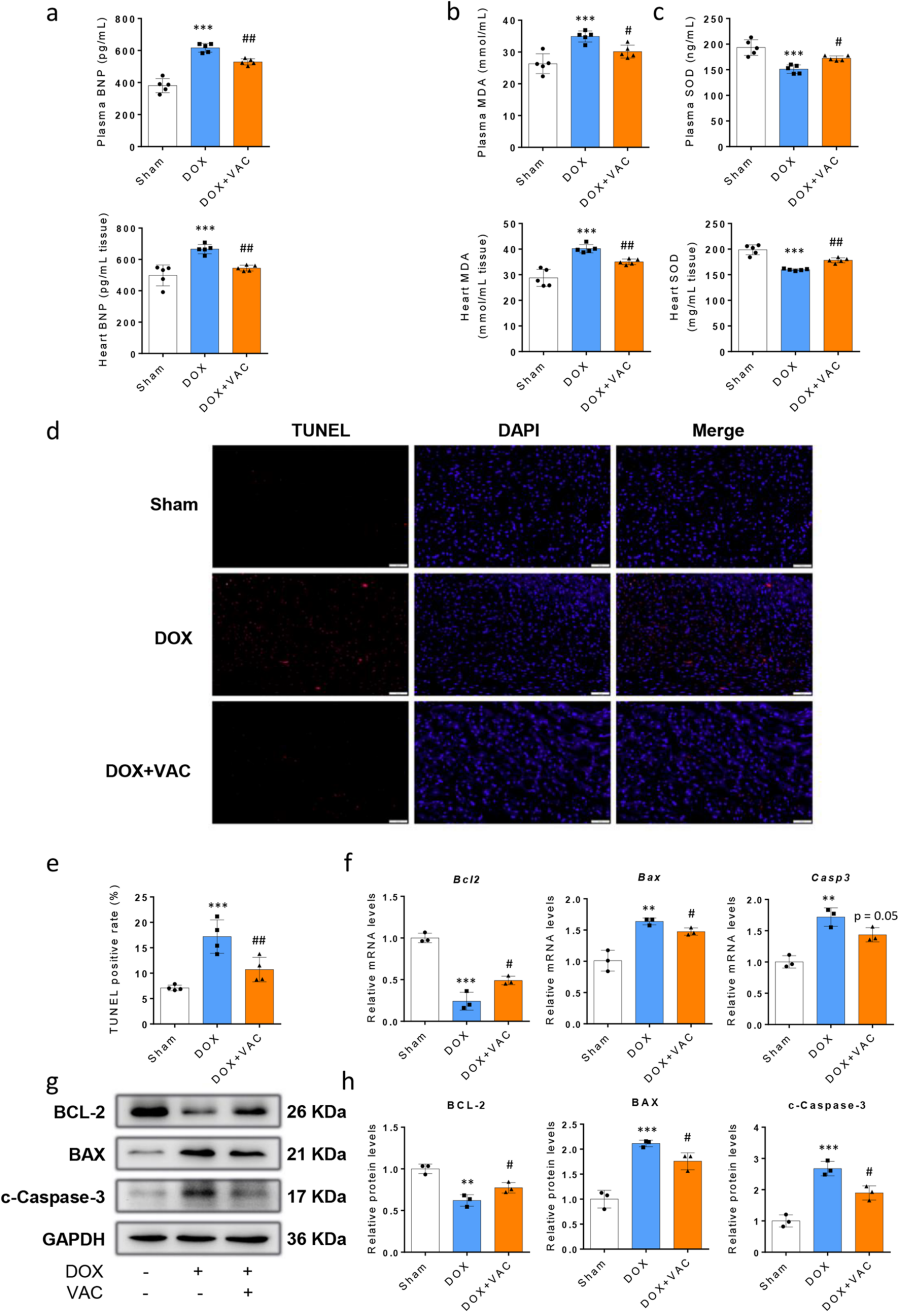
Vaccarin ameliorates oxidative stress-mediated apoptosis in DOX-induced cardiotoxicity. **a** B‐type natriuretic peptide (BNP) levels in plasma and heart homogenate were examined by ELISA in the three groups of mice (*n* = 5). **b**-**c** Concentration of the malondialdehyde (MDA) and activity of superoxide dismutase (SOD) in plasma and heart homogenate were detected by the MDA and SOD kit respectively (*n* = 5). **d**-**e** Cardiomyocytes apoptosis was examined by TUNEL staining (red). The blue fluorescence indicates the cell nucleus stained by DAPI. Bar graph shows the percentage of TUNEL positive cells per field at × 200 magnification (*n* = 4). **f** Quantitative Real-time PCR (Q-PCR) detected the gene expression of Bcl2, Bax, and casp3 (*n* = 3). **g**-**h** Representative Western blot image and semi-quantification results showed the protein level of BCL-2, BAX, and cleaved-CASPASE-3. GAPDH is set as the internal control (*n* = 3). The experiment was repeated 3 times. Data are represented as the mean ± SEM (*t* test, ***p < 0.001 between groups of DOX and Sham. ^#^*p* < 0.05, ^##^*p* < 0.01, and ^###^*P* < 0.001 between groups of DOX + VAC and DOX)

Brain natriuretic peptide (BNP) test results in mouse plasma and heart homogenate also testify that VAC improves the heart function in DOX-induced cardiotoxicity (Fig. 2a). As oxidative stress is widely accepted as a major factor of DOX-induced cardiotoxicity [2], we next investigated whether VAC inhibit oxidative stress and evaluated its antioxidant capacity in DOX-induced cardiotoxicity. Indeed, we found that the level of malondialdehyde (MDA), one of the biological markers for oxidative stress, significantly increased in DOX alone group compared to the sham control in plasma and heart. Meanwhile, the activity of superoxide dismutase (SOD) decreased compared to the sham. Strikingly, additional VAC administration markedly decreased MDA and increased SOD levels in both plasma and heart compared to the DOX alone group (Fig. 2b-c). These data correlated with the TUNEL assay, which exhibited the cell apoptosis of DOX-induced cardiotoxicity. As shown in Fig. 2d-e, the apoptosis index (TUNEL-positive cells/field) significantly reduces in the group received DOX + VAC when compared to the group with DOX alone.

Moreover, previous studies revealed that following exposure to doxorubicin, there is initial upregulation of the expression of the anti-apoptotic proteins, Bcl-XL, Bcl-2, followed by decreases in their expression, and increasing the expression ratio of Bcl-2/Bax can protect against doxorubicin-induced cardiotoxicity [24]. In addition, the activity of caspase-3 increases in DOX-induced cytotoxicity [25]. To evaluate the effects of VAC on these apoptosis markers, we detected the gene expression by RT-PCR and found a dramatic decrease in the mRNA expression of *Bcl2*, an increase in the expression of *Bax* and *Casp3* (encodes caspase-3) in DOX treated heart, as compared with the sham control. As shown in Fig. 2f, the additional use of VAC significantly inhibited the expression of *Bax* and *Casp3*-, and induced the expression of *Bcl2*. Meanwhile, we examined the protein levels of these three molecules. As expected, VAC suppressed the pro-apoptotic BAX, and activated Caspase-3 (cleaved), but increased the anti-apoptotic BCL-2 protein level (Fig. 2g-h).

### Vaccarin Specifically Inhibits the Activation of p38 MAPK Pathway in DOX-induced Cardiotoxicity

**Fig. 3:**
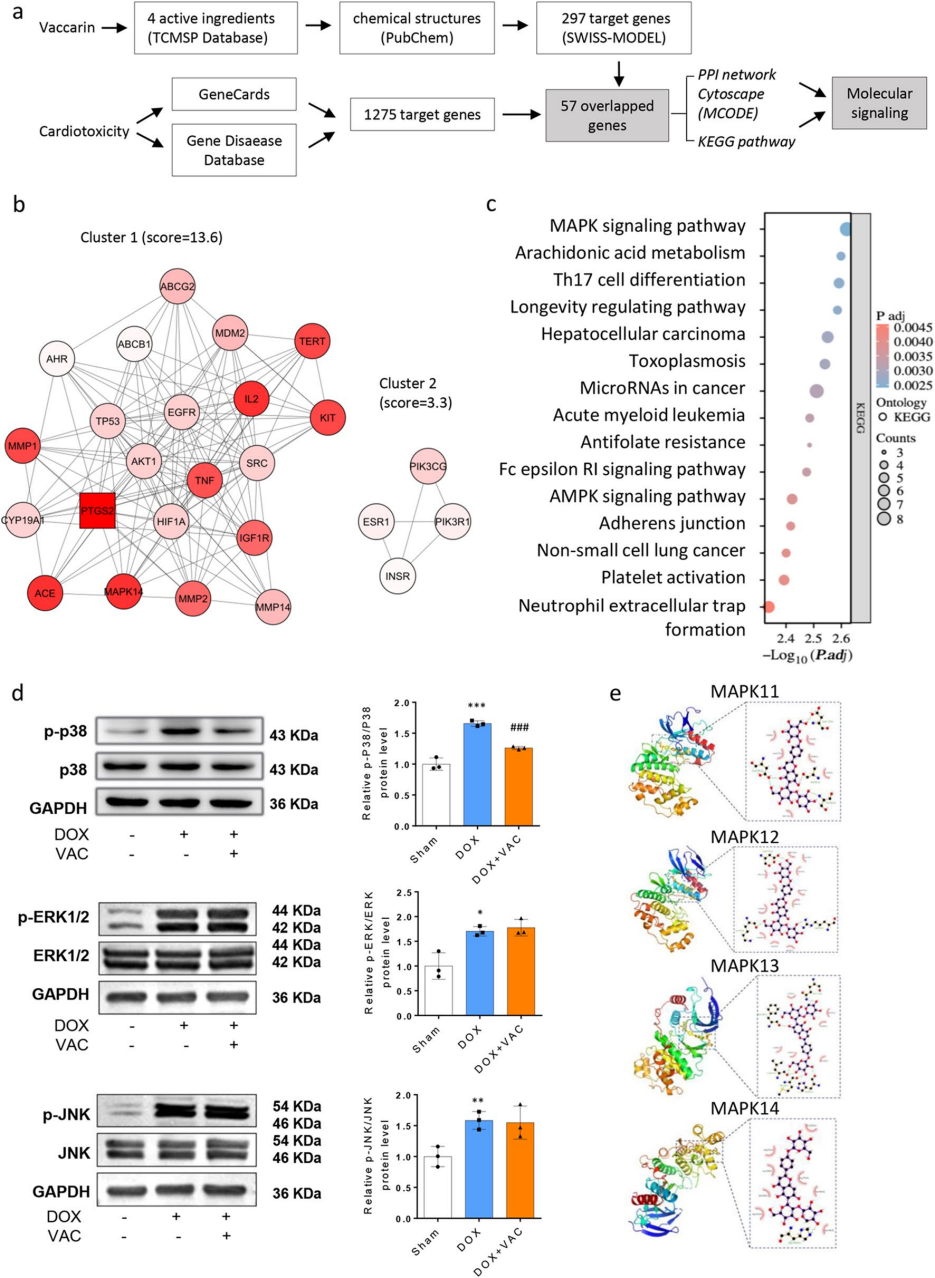
Vaccarin specifically inhibits the activation of p38 MAPK pathway in DOX-induced cardiotoxicity. **a** The working flow of bioinformatics to identify potential genes targeted by vaccarin and cardiotoxicity and related pathways. **b** The PPI network of the 57 target genes identified two clusters by using STRING-Cytoscape (MCODE). 21 hubs was included in Cluster 1. Node shapes indicate the cluster status of the nodes. Square: seed (highest scoring node in the cluster). Circle: clustered. A range from white to red indicates the MCODE computed node score (lowest to highest, respectively).**c** KEGG pathway analysis identified 15 pathways associated with the 57 input genes, among which MAPK signaling pathway is ranking the top according to adjusted *P*. Value. **d** Whole and phosphorylated protein levels of p38, ERK1/2, and JNK in heart samples of the three groups were assessed by Western blot. Representative image and semi-quantification results are shown (*n* = 3). Data are represented as the mean ± SEM *(t* test, **p < 0.01, and ***p < 0.001 between groups of DOX and Sham. ^###^*p* < 0.001 between groups of DOX + VAC and DOX.) **e** The molecular docking of vaccarin with p38 MAPK isoforms. Prediction of protein binding sites using POCASA 1.1 and docking using AutoDock Vina1.5.6

To explore the potential molecular mechanism through which VAC inhibits DOX-induced cardiotoxicity, we at first retrieved active ingredients of VAC and then acquired 297 potential target genes using SwissTargetPrediction (Table 1). The detail steps were introduced in the methods, and a flowchart was presented in Fig. 3a. Next, we retrieved 1280 genes those associated with cardiotoxicity by screening the GeneCards (https://www.genecards.org/) and the DisGeNetdatabases (https://www.disgenet.org/). By matching the VAC targets and the cardiotoxicity associated genes, 57 overlapping genes were harvested (Table. S1). Next, the 57 targets were uploaded to STRING, a biological database to predict protein–protein interactions (PPI). The PPI network was then analyzed by Cytoscape-MCODE (Molecular Complex Detection), two clusters (highly interconnected regions) were found (Fig. 3b). A higher score is given to those nodes whose neighbors are more interconnected. Within 21 hubs in Cluster 1, PTGS2, ACE, IL2, MAPK14, and KIT were the top five high score nodes in the densely connected regions (Table. S2). Meanwhile, 15 KEGG pathways were identified from the 57 input genes, with the MAPK signaling pathway ranking the first (Fig. 3c). This suggested that the predicted complex in Cluster 1 might be involved in regulating MAPK pathway, although the direction of regulation is unknown.

**Table 1:** Active ingredients in vaccarin

Mol ID	Molecule Name	Pubchem CID	Canonical SMILES
MOL000449	Stigmasterol	5280794	CCC(C = CC(C)C1CCC2C1(CCC3C2CC = C4C3(CCC(C4)O)C)C)C(C)C
MOL002322	Isovitexin	162350	C1 = CC(= CC = C1C2 = CC(= O)C3 = C(O2)C = C(C(= C3O)C4C(C(C(C(O4)CO)O)O)O)O)O
MOL008908	Segetalin B	10345235	CC1C(= O)NCC(= O)NC(C(= O)NC(C(= O)NC(C(= O)N1)CC2 = CNC3 = CC = CC = C32)C)C(C)C
MOL000098	Quercetin	5280343	C1 = CC(= C(C = C1C2 = C(C(= O)C3 = C(C = C(C = C3O2)O)O)O)O)O

Indeed, the p38 MAPK pathway, together with various signaling cascades such as JNK, ERK, regulates the balance between cell survival and cell death, and p38-MAPK pathway has been reported to mediate the inhibition of VAC on ox-LDL-induced HUVEC EndMT [14], and on titanium particle-induced osteolysis [27]. However, how VAC functions on cardiomyocytes, especially on cardiomyocytes under DOX-treated condition, is completely unknown. To explore whether p38-MAPK pathway plays a role in mediating the protection of VAC on DOX-induced cardiotoxicity, we examined the protein level of p38, ERK1/2, JNK and their endogenous phosphorylated forms. We found that DOX treatment significantly increased phosphorylated p38, ERK1/2 and JNK levels in heart with compared to sham, and DOX plus VAC specifically inhibited phosphorylated p38 level but did not change p-ERK1/2, and p-JNK (Fig. 3d).

We then used molecular docking analysis and measured the binding affinity (Docking Free energy) and amino acid interactions of VAC and p38 isoforms (Fig. 3e). The details regarding the number of hydrogen bonds shared with the amino acid/nucleotide residues at the active site regions of target proteins are shown in Table 2. The results suggest that VAC and p38 isoforms have a great binding efficiency ranging from -7.7 to -9.7 kcal/mol, which may impair the activation of p38 [26].

**Table 2:** Amino acid residues participating in hydrogen bond interaction in p38 isoforms with vaccarin

Isoform	Binding Engery (kcal/mol)	Residue form H-bonds	Distance (Å)	hydrophobic interactions
p38α	-7.7	His199	2.95	Tyr258、Trp197、Asn196、Met198、Ser252、Ile250、Leu195、Ala255
p38β	-9.7	Asp112	2.83	Trp35、Val38、Lys53、Glu71、Leu167、Val30
		Ala34	2.97	
		Ser56	2.99	
		Asp168	2.71	
			2.99	
p38γ	-9.0	Arg70	2.87	Thr114、Lys118、Val33、Asp171、Val41、Phe67、Leu58、Tyr59、Lys56、Leu170、Phe111
		Lys57	2.98	
		Asp115	2.86	
p38δ	-8.2	Gln111	2.99	Asn159、Glu160、Pro108、Gly86、Leu88、Lys50
		His81	2.77	
		Leu87	2.72	
			2.74	
		Phe109	2.91	
		Met79	2.84	

### Vaccarin Pretreatment Prevents DOX-induced Cell Death and Shrinkage in H9c2 cell, but the p38 MAPK Agonist Anisomycin Reverses this Effect

**Fig. 4:**
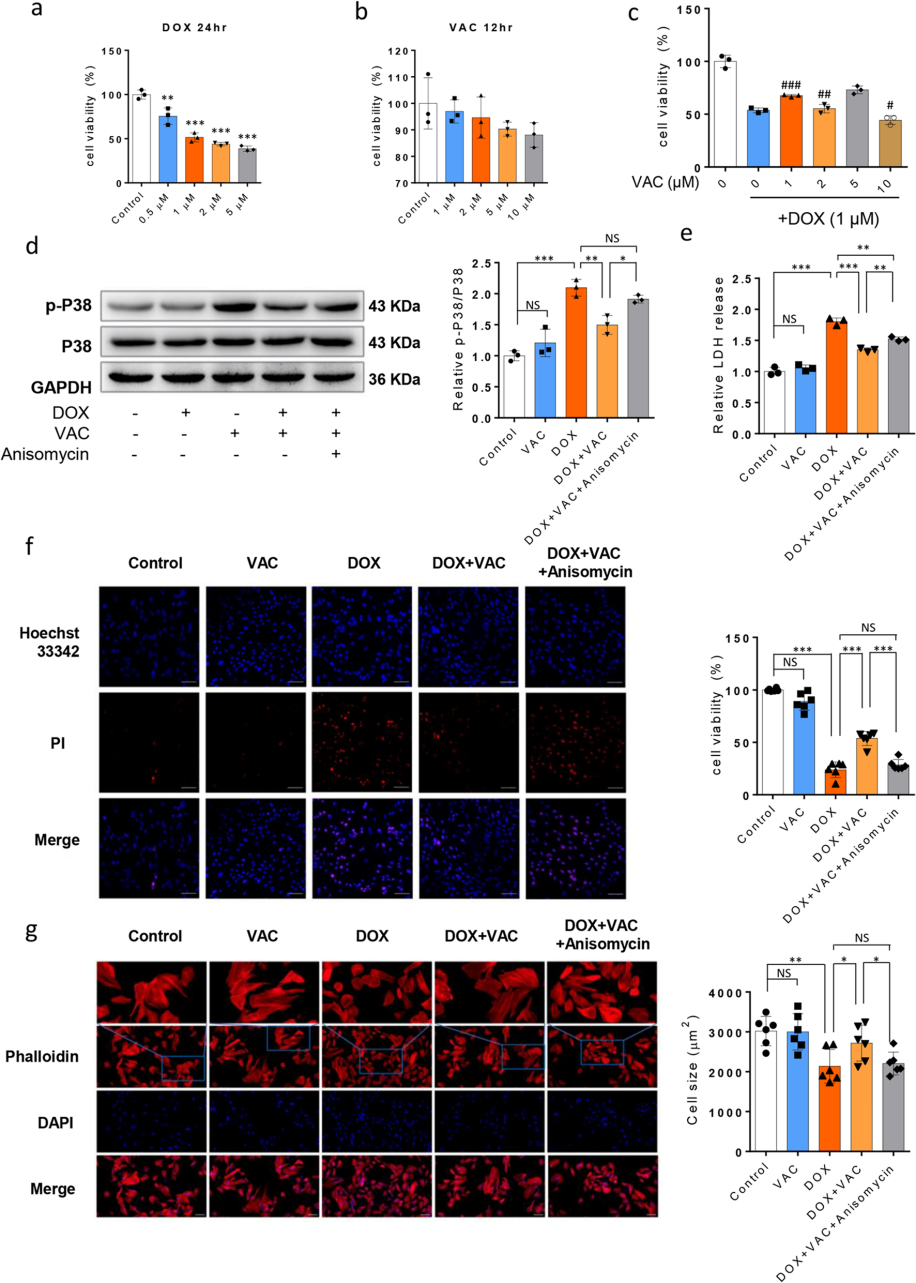
Vaccarin alleviates DOX-induced H9c2 cell death by inhibiting p38 activation. **a** H9c2 cells were treated with DOX at different concentrations for 24 h. **b** H9c2 cells were treated with VAC at different concentrations for 12 h. **c** H9c2 cells were pre-incubated with different doses of VAC for 12 h and then stimulated with DOX for 24 h. VAC alleviates DOX-induced H9c2 cell death and has the maximal effect at 1 μM. Cell viability was determined by CCK-8. **d** Whole and phosphorylated protein levels of p38 in cultured H9c2 cells were assessed by Western blot. Five groups of cell extracts were prepared after stimulation with DOX (1 μM) for 24 h with or without 12 h VAC pre-incubation (1 μM), plus anisomycin (10 μM), an agonist of p38 MAPK. Representative image and semi-quantification results showed. **e** Release of LDH in H9c2 cells after stimulations was examined by LDH assay kit. **f** Hoechst 33,342 (blue) / Propidium Iodide (PI, red) double staining demonstrate the cell viability after stimulation with DOX or/and Vaccarin, plus anisomycin. Images were detected with a fluorescence microscope. Magnification =  × 100. **g** Representative images indicate cytoskeleton of H9c2 cells after the treatments by CellMask™ Deep Red Actin Tracking. The blue fluorescence indicated the cell nucleus stained by DAPI. Bar graph shows the cell size measured via fluorescence microscope. Magnification =  × 100 (First row, Magnification =  × 300). The experiments were repeated 3 times. Data are represented as the mean ± SEM. (One-way Anova Tukey’s multiple comparisons test, *p < 0.05, **p < 0.01, and ***p < 0.001.NS, no significance.)

To further evaluate the involvement of the p38 MAPK signaling pathway in the protective actions of VAC on DOX-induced cardiotoxicity, we set up an in vitro model using embryonic cardiomyocyte cell line H9c2, which is commonly used in cardiotoxicity analyses of new drugs [28]. First, we used CCK8 assays to determine the effects of DOX and VAC on cell viability of H9c2 cells. As shown in Fig. 4a, DOX caused cell death after 24 h of treatment, and this effect was dependent on the drug concentration. In line with previous reports [29], we used 1 µM of DOX for 24 h to induce cardiotoxicity. Since VAC has never been used on H9c2 cells before, we began testing various concentrations (1, 2, 5, 10 µM) of VAC on cells for 12 h and found that VAC treatment alone did not affect cell survival (Fig. 4b). Next, to explore the effect of VAC on cellular damage induced by DOX in H9c2 cells, we selected a 12-h pre-incubation period for VAC prior to DOX exposure, consistent with previous in vitro models of DOX-induced cardiotoxicity [11, 30]. We observed that VAC alleviated DOX-induced H9c2 cell death, with the maximal effect seen at 1 μM (Fig. 4c). Therefore, the VAC concentration at 1 μM was used for the following experiments. Building upon our findings in vivo, we examined the protein level of phosphorylated p38, ERK1/2, and JNK, in the in vitro system under DOX, VAC stimulation, or both conditions. As expected, we found that VAC specifically inhibited DOX-induced p-p38 but did not alter p-ERK1/2, and p-JNK (Fig. S1).

Next, through the introduction of supplementation with anisomycin (10 μM), an agonist of p38 MAPK, we observed that the protective effects of VAC against DOX-induced cell death (Fig. 4f) and cell atrophy (Fig. 4g) were significantly diminished by anisomycin, concomitant with the activation of p38 (Fig. 4d). Furthermore, LDH is rapidly released into the cell culture supernatant when cell membrane is damaged, which is a key feature of cells undergoing apoptosis, necrosis, and other forms of cellular damage [31]. As shown in Fig. 4e, the relative LDH release also provided the evidence that VAC protects against DOX-induced cell death via p38 MAPK signaling pathway.

### Vaccarin Alleviates DOX-induced H9c2 cell Apoptosis by Restoring Mitochondrial Membrane Potential and Inhibiting ROS Induction via p38-MAPK Pathway

**Fig. 5:**
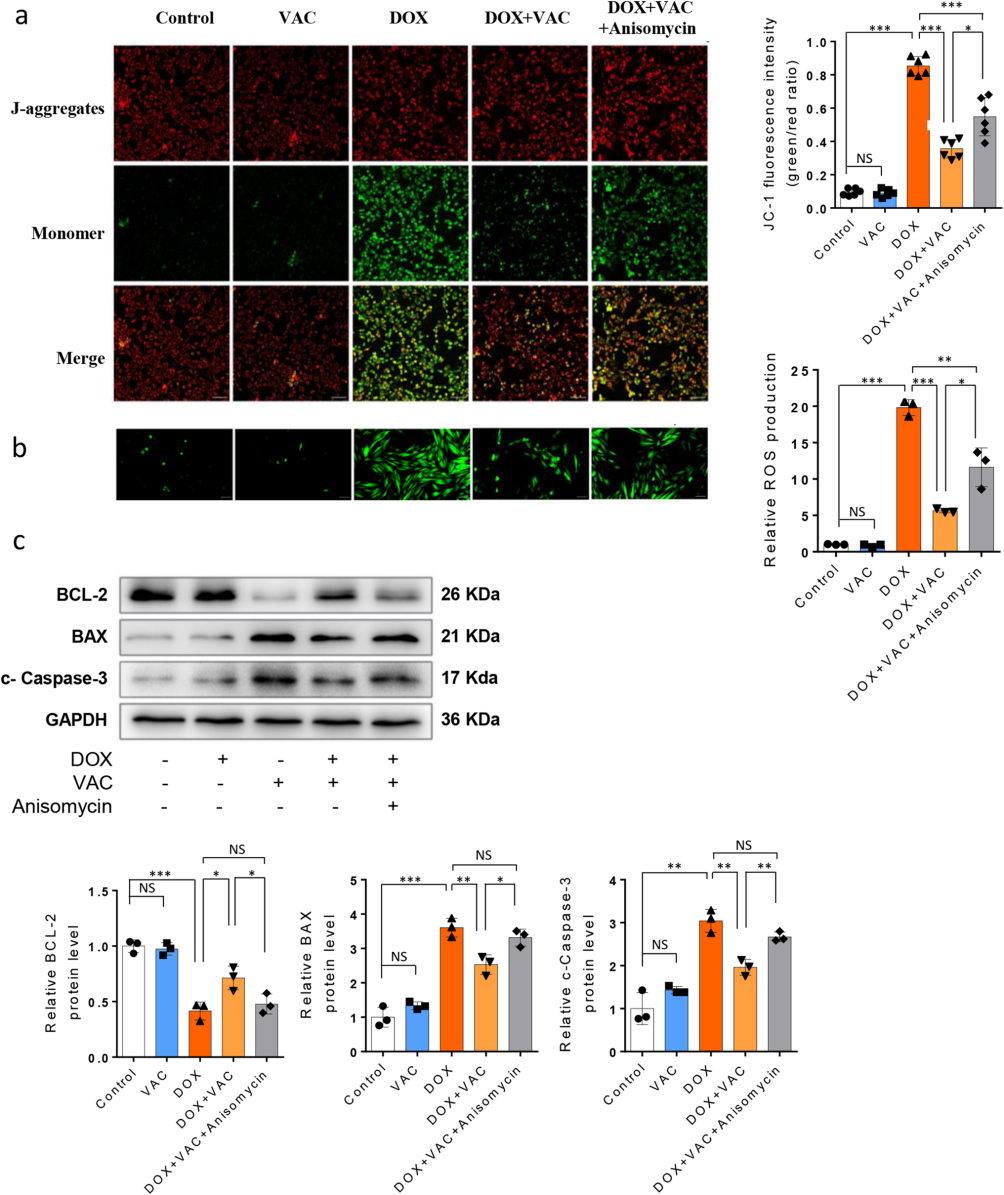
Vaccarin alleviates DOX-induced mitochondrial depolarization, ROS production and cell apoptosis in H9c2 cells, effects are blocked by P38 agonist anisomycin. **a** JC-1 staining show the mitochondrial health in H9c2 cells after stimulation with DOX or/and Vaccarin, plus anisomycin. At higher mitochondrial concentrations or higher membrane potentials, JC-1 dye monomer forms red fluorescent "J-aggregates". The J-aggregates exhibit a broad excitation spectrum and an emission maximum at ~ 590 nm (red). At lower internal mitochondrial concentrations or low membrane potential, the JC-1 dye is present as monomers, exhibiting an emission of 529 nm (green). Bar graph show the green/red fluorescence intensity ratio, which indicates the mitochondrial depolarization (*n* = 3). Magnification =  × 40. Scale = 200 μm. **b** ROS levels in H9c2 cells were measured with CellROX Green reagent after stimulations. The green fluorescence indicates the ROS production in cells. Magnification =  × 100. Bar graph show the semi-quantification results. **c** Representative Western blot image and semi-quantification results showed the protein level of BCL2, BAX, cleaved-CASPASE-3. GAPDH is set as the internal control. The data represent the mean ± SEM (*n* = 3). (One-way Anova Tukey’s multiple comparisons test, *p < 0.05, **p < 0.01, and ***p < 0.001)

As mentioned earlier, VAC reduces DOX-induced oxidative stress and cell apoptosis in the mouse heart. VAC also restored DOX-impaired SOD level, one of the first-line defense antioxidants in plasma and heart tissues. VAC’s inhibition of p38-MAPK pathway are responsible for rescuing the DOX-induced imbalance between the generation of oxidants and the local antioxidative defense. We then examined VAC’s anti-apoptotic effects in vitro with or without the administration of anisomycin. Using JC-1 staining, a fluorescent cationic carbocyanine dye that accumulates in mitochondria in a potential-dependent manner, forming J aggregates in healthy cells (red) and diffusing into a monomeric state upon depolarization in apoptotic cells (green), we observed that VAC significantly decreases the DOX-induced mitochondrial depolarization, and anisomycin partially reverse this suppression (Fig. 5a). Meanwhile, we examined the ROS levels under those stimulation conditions and found that VAC inhibited DOX-induced ROS production, with the inhibition weakened by supplementation with anisomycin (Fig. 5b). A strong correlation between ROS production and mitochondrial membrane potential (ΔΨm) has been confirmed [32]. In cases of mitochondrial disorders associated with the dysfunctions of the respiratory chain components, a decrease in ΔΨm is observed concurrently with an increase in ROS production, ultimately leading to apoptotic cell death. Futhermore, we examined the protein levels of BCL-2, BAX, and active Caspase-3. We observed that the elevation of the pro-survival protein BCL-2 and the reduction of the pro-apoptotic proteins BAX and caspase-3, which occurred because of VAC pre-treatment, were attenuated by anisomycin (Fig. 5c). These results suggest that VAC ameliorates DOX-induced mitochondrial dysfunction, subsequently reducing cell apoptosis through inhibiting p38 MAPK pathway.

## Discussion

Finding safe and effective treatments for DOX-induced cardiotoxicity is of vital importance. In this study, we first identified that Vaccarin improves heart function and alleviates cardiac atrophy in a mouse DOX cardiomyopathy model. Additionally, VAC ameliorates ROS generation, mitochondrial depolarization, and apoptosis in cardiomyocytes in vivo and in vitro following doxorubicin exposure, and these effects are mechanistically linked to the specific inhibition of the p38-MAPK pathway. Administration of p38 MAPK agonist anisomycin attenuates the protective effects of VAC. Our findings demonstrate that VAC ameliorates DOX-induced cardiotoxicity by inhibiting p38 MAPK pathway, suggesting that VAC may serve as a potential therapeutic candidate to reduce the risk of DOX cardiomyopathy.

A cumulative dose of DOX ranging from about 15 to 36 mg/kg can lead to cardiotoxicity in mice and rats [19]. In our study, we administered 20 mg/kg, divided into 5 mg/kg doses via I.P. once a week for four consecutive weeks. We observed a decrease of over 20% in left ventricle ejection fraction (LVEF), which is indicative of impaired cardiac function. The administration of VAC in a daily regimen for 4 weeks leads to the recovery of LVEF, as well as other echocardiographic parameters of left heart function, and ameliorates heart atrophy. These results first demonstrates the beneficial effects of VAC on a DOX-induced cardiotoxicity mouse model. Of note, the in vivo protections are achieved through daily I.P. of VAC at 30 mg/kg, the same dose that has been reported to significantly alleviate hypertension, cardiovascular remodeling and nephropathy in renovascular hypertensive rats [5]. In the former study, a lower dose of VAC (10 mg/kg) was applied, and although it showed statistically significant improvement, the outcome was far less than that achieved with the dose of 30 mg/kg. Therefore, we chose to use VAC at 30 mg/kg per day for the in vivo model and observed significant improvement without detectable side effects within our observation window. Of note, in the in vitro system using H9c2 cells, we found that higher dose of VAC (at 5 µM) pre-treatment does not improve DOX-induced H9c2 cell injury, but on the contrary, exacerbates cell death. So far, this dose have been used in various in vitro system, such as on HUVECs (at 5 µM for 12 h and 16 h) [14], HMEC-1 cells (at 2.15–17.2 µM for 48 h) [6], 3T3-L1 cells (at 3.44–13.76 µM for 24 h) [33], Caco-2 cells (human colonic adenocarcinoma cell line, at 5 µM for 48 h) [10], and BMMs (bone marrow macrophages, at 4–32 µM for 48 h) [27], however, no cell cytotoxicity has been reported. Given that this is the first time of VAC being used on H9c2 cells, this finding has drawn our attention to the potential resistance of cardiomyocytes, particularly H9c2 cells, to high doses of VAC, when applied under DOX-exposure conditions. Hence, in the future, it will be necessary to explore additional doses and observation timelines, or consider an alternative cardiomyocyte cell line, such as HL-1 cells, when investigating the efficacy and safety of VAC in alleviating DOX-induced cardiotoxicity in vitro.

Almost every group of flavonoids are rich in antioxidant activity [34]. Therefore, identify VAC-associated pathways in ameliorating DOX-induced cardiomyocytes apoptosis, can help understand disease mechanisms and improve diagnostics and develop efficient treatments. Much have been learned about the mechanisms of therapeutic effects of doxorubicin on tumor cells are different from those of the mechanisms of its cardiotoxicity [1]. DOX exerts its antitumor effects by inserting into DNA, resulting in double-stranded DNA breaks (DSB), and intercepting DNA topoisomerase II activity. In contrast, DOX induces oxidative stress, leading to cardiomyocyte apoptosis via downregulation of Bcl-2, upregulation of Bax, and activation of caspase-3. Mechanistically, many signaling pathways, including PI3k/Akt/Mtor [1, 35], MEK/ERK [36], and JNK [37], have been demonstrated involving into the process. Among the intracellular signaling molecules, p38 MAPK is one of the most studied target molecules for triggering DOX-induced cardiotoxicity [38–40]. In this study, we found phosphorylation levels of p38, JNK and ERK increase in mouse heart and H9c2 cells upon DOX treatment, and VAC partially inhibits p38 activation*,* leading a decrease of pro-apoptotic regulatory proteins, and an increase of anti-apoptotic protein. The use of anisomycin diminishes the anti-apoptotic effects of VAC. Although anisomycin has been wildly used as a selective activator of p38, it also activates JNK [41]. Our in vivo and in vitro results showed that VAC only inhibits p38, but alters neither JNK nor ERK. Therefore, we believe that anisomycin antagonize VAC’s effects via the activation of p38. Collectively, we demonstrated for the first time that VAC protects against DOX-induced cardiotoxicity via p38 MAPK signaling pathway.

The four isoforms of p38 MAPK are encoded by distinct genes, which are p38α (*Mapk14*), p38β (*Mapk11*), p38γ (*Mapk12*), and p38δ (*Mapk13*) [42]. Among them, p38α is activated in response to numerous cellular stresses and plays a role in regulating the differentiation and/or survival of various cell types in vitro, including skeletal muscle cells and cardiomyocyte [43]. In the protein–protein interaction networks, we identified *Mapk14* as one of the top five regulators in predicted protein complex that actively involved in the biological effects of VAC and cardiotoxicity, along with *Ptgs2* (COX-2), *Ace*, *Il2*, and *Kit* (c-kit). Molecular docking analysis is capable to predict the binding-conformation of small molecule ligands to the appropriate target-binding site [44, 45]. Using this strategy, we found the binding efficiency of VAC and p38 isoforms ranging from -7.7 to -9.7 kcal/mol is greater than existing FDA-approved molecules (-5.18 to 6.66) and even slightly better than the top known p38-inhibitors (-6.68 to -7.81) [46, 47]. By impairing the capacity of p38 to bind and phosphorylate its substrates, VAC diminishes p38 MAPK signaling cascade, including protein kinases, phosphatases, cell-cycle regulators, and transcription factors [48]. High affinity is typically linked to a lower dosage requirement [49], suggesting VAC could be a promising candidate as a p38 inhibitor to protect against cardiotoxicity. Of note, drug resistance frequently occurs in anti-cancer therapy, and studies revealed that the p38 MAPK signaling pathway is involved in doxorubicin-induced drug resistance [50]. Therefore, in addition to alleviating DOX-induced cardiotoxicity, supplementation with VAC could provide a new chemotherapeutic option for overcoming drug resistance in cancer treatment.

Our results demonstrated VAC’s inhibition of p38-MAPK pathway are responsible for rescuing the DOX-induced imbalance between the generation of oxidants and the local antioxidative defense. DOX-induced p38 activation can inhibit the anti-apoptotic role of Bcl-2 by phosphorylating Bcl-2 and regulating Bax mitochondrial translocation. This results in ΔΨm loss and sensitizes cardiomyocytes to apoptosis [51, 52]. VAC significantly reduces DOX-induced mitochondrial depolarization and ROS generation. Unlike its almost complete reversal of VAC's effects on Bcl-2/Bax and caspase-3, anisomycin only partially reversed VAC's inhibition of mitochondrial depolarization and ROS production. This inconsistency suggests the possibility of other pathways being involved in VAC's protection against mitochondrial depolarization and ROS production. It has been demonstrated that one of the beneficial effects of flavonoids on cardiovascular diseases is their ability to block the rise of intracellular calcium, exerting their antiplatelet effect and inducing vasodilation [53]. Dysregulation of calcium homeostasis plays an important role in the pathogenesis of DOX-induced cardiotoxicity [54], and reducing high calcium levels could alleviate depolarization of the mitochondria, followed by a restoration of ATP synthesis, thereby reducing cell death [55]. However, whether VAC is capable of inhibiting DOX-induced intracellular calcium overload and subsequently reducing mitochondrial depolarization and ROS production is completely unknown. Future work is essential to fill this gap, and understanding this will contribute to advancing the knowledge of VAC therapy for DOX-induced cardiotoxicity and other cardiovascular diseases.

There are several limitations in our study. First, healthy animals were used to establish the DOX-induced cardiotoxicity models. To consolidate the VAC therapy for DOX-induced cardiotoxicity and provide adequate similarity to susceptible human with neoplasms, future work is required to concurrently induce DOX-induced cardiomyopathy alongside modeling the tumor process. Of note, recent publications reported that active components of VAC themselves played significant inhibitory effects on lung cancer [56] and colorectal cancer [57]. These types of cancer are inherently resistant to DOX, which can cause cardiotoxicity at higher doses. These findings have provided us with future directions for selecting cancer models. The second limitation of this study is our incapacity to expand our in vivo research by using elevated VAC concentrations to guarantee both effectiveness and safety, potentially constraining the broader applicability of our findings.

In conclusion, this study, for the first time, demonstrates that VAC alleviates DOX-induced cardiotoxicity. By inhibiting the activation of the p38-MAPK pathway, VAC significantly recovers heart functions and reduces tissue oxidative stress and cell apoptosis *through recovering mitochondrial membrane potential and inhibiting ROS induction*. These findings provide a promising natural antioxidant to protect against DOX-induced cardiotoxicity, potentially increasing patient survival and improving the quality of life for cancer survivors.

## Supplementary Information

Below is the link to the electronic supplementary material.

Supplementary file1 (XLSX 39 KB)

Supplementary file2 (XLSX 33 KB)

Supplementary file3 (PNG 33 KB)

High resolution image (TIF 8.79 kb)

## Data Availability

The authors declare that the data supporting the findings of this study are available within the paper and its Supplementary Information files. Should any raw data files be needed in another format they are available from the corresponding author upon reasonable request.

## Abbreviations

DOX: Doxorubicin
VAC: Vaccarin
LVEF: Left ventricular ejection fraction
LVFS: Left ventricular fractional shortening
LVPWd: Left ventricular posterior wall thickness in at diastole
LVPWs: Left ventricular posterior wall thickness in at systole
LVEDs: Left ventricular end-systolic dimension
LVEDd: Left ventricular end-diastolic dimension
ΔΨm: Mitochondrial membrane potential

## Declarations

### Animal Studies

The experimental procedures were ethically approved by the Harbin Medical University Committee on Animal Care and were carried out in accordance with the Regulations for the Administration of Affairs Concerning Experimental Animals approved by the State Council of China.
