# Supplementary figures and images for: Vaccarin Ameliorates Doxorubicin-Induced Cardiotoxicity via Inhibition of p38 MAPK Mediated Mitochondrial Dysfunction
Source: J Cardiovasc Transl Res. 2024 Jun 17;17(5):1155–71. doi: 10.1007/s12265-024-10525-7 (PMC11519163; doi:10.1007/s12265-024-10525-7)

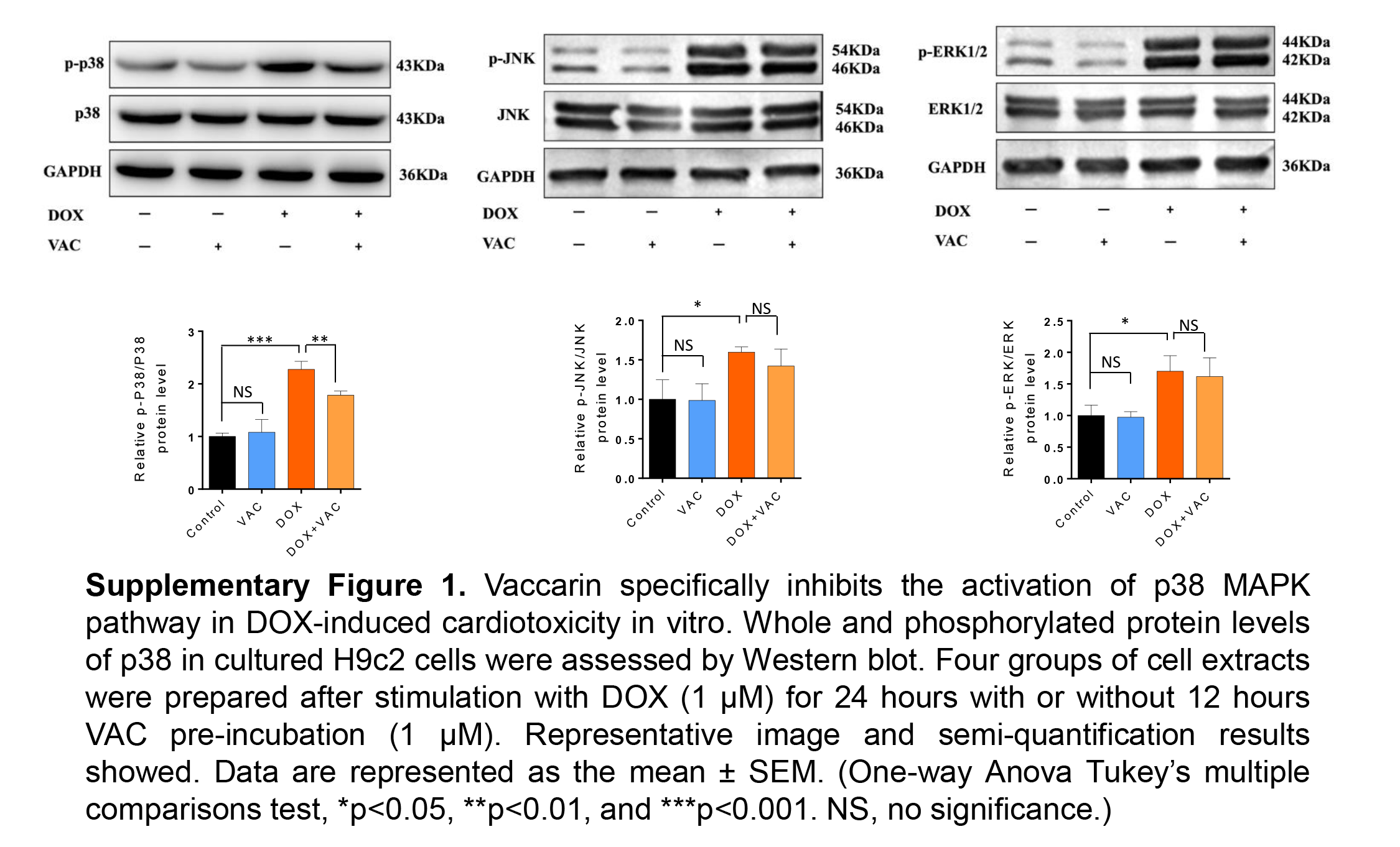

Supplement: Supplementary file 3 — Supplementary file3 (PNG 33 KB) [file 12265_2024_10525_Fig7_ESM.png]

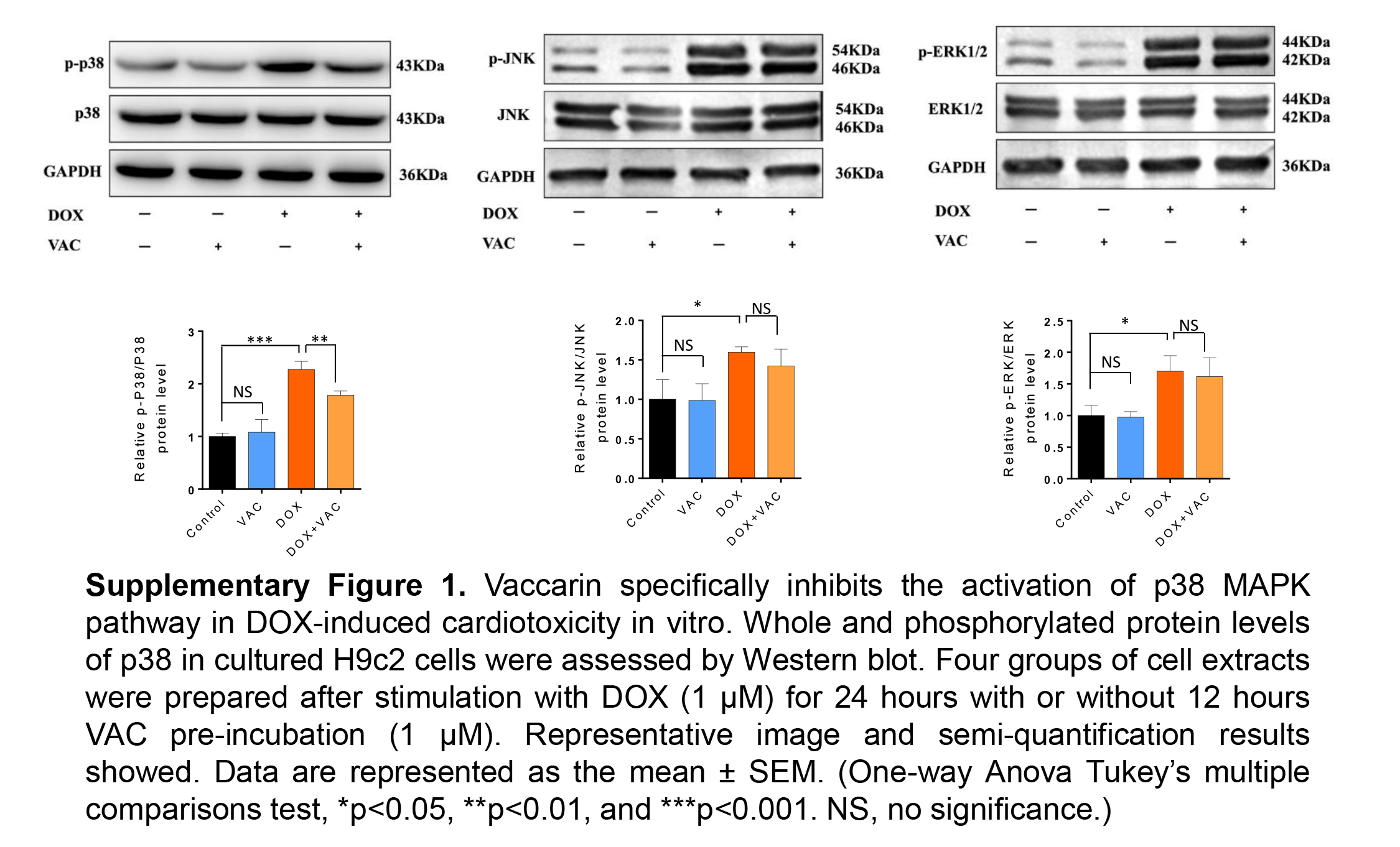

Supplement: Supplementary file 4 — High resolution image (TIF 8.79 kb) [file 12265_2024_10525_MOESM3_ESM.tif]
